# EHMTI-0072. Anodal transcranial direct current stimulation alleviates pain in trigeminal neuralgia

**DOI:** 10.1186/1129-2377-15-S1-E21

**Published:** 2014-09-18

**Authors:** M Obermann, V Bude, D Holle, S Naegel, T Hagenacker, HC Diener, Z Katsarava

**Affiliations:** 1 Department of Neurology, University Hospital Essen, Essen, Germany

## Introduction

TN usually leads to paroxysms of short lasting but very severe pain. Between the attacks the patient is usually asymptomatic, but a constant dull background pain may persist. Carbamazepine is currently the drug of first choice in the treatment of TN. It is efficient in 70-80% of patients but often associated with severe adverse effects such as drowsiness, confusion, nausea and ataxia, which may require discontinuation of medication. Surgical interventions may not be suitable for all patients and waiting for the procedure can be agonizing. Therefore different non-invasive treatment options are indispensable.

## Aims

To investigate the efficacy of transcranial direct current stimulation (tDCS) of the primary motor cortex on pain and trigeminal nociceptive processing in subjects with classical trigeminal neuralgia (TN).

## Methods

Seventeen subjects with TN were recruited in the study. Patients stimulated daily for 20 minutes over two weeks using anodal (1mA) or sham tDCS in a randomized cross-over design. Primary outcome variable was pain intensity on a verbal rating scale (VRS). VRS and attack frequency were assessed daily for one month before and after tDCS using an individual patient diary. The impact on trigeminal pain processing was assessed with pain-related evoked potentials (PREP) and the nociceptive blink reflex (nBR) following electrical stimulation on both sides of the forehead (V1) before and after tDCS. All patients gave written informed consent prior to study inclusion. The study was approved by the Ethics committee of University of Duisburg-Essen medical faculty.

## Results

Anodal tDCS reduced pain intensity more effectively than sham stimulation after two weeks of treatment. The attack frequency reduction was not significant. PREP showed an increased N2 latency and decreased peak-to-peak amplitude after anodal tDCS. No severe adverse events were reported. Patients with concomitant chronic background pain do not seem to benefit from tDCS as described previously for medical therapy and surgical intervention.

## Conclusions

Daily anodal tDCS over two weeks ameliorates trigeminal pain in TN. It may become a valuable treatment option for patients unresponsive to conventional medical treatment or on wait for surgical procedures. International, multicenter, randomized controlled trials are needed with higher patient numbers before a definite recommendation can be proposed.

No conflict of interest.

